# False Positive Troponin I Rendering Two Admissions for “Recurrent Acute Myopericarditis”

**DOI:** 10.2174/1874192401812010055

**Published:** 2018-06-29

**Authors:** Rita Marinheiro, Pedro Amador, Leonor Parreira, Quitéria Rato, Rui Caria

**Affiliations:** Cardiology Department, Centro Hospitalar de Setúbal, Setúbal, Portugal

**Keywords:** Troponin I, Assay, False positive, Myopericarditis, Alkaline phosphatase, Echocardiography

## Abstract

**Introduction:**

Since there are many manufacturers of cardiac troponin I assays, the true incidence of a false positive result is unknown. The authors report a case of an 18-year-old patient with previous history of recurrent myopericarditis and admitted to hospital twice again with a suspicious of myopericarditis.

**Conclusion:**

Troponin I was found to be a false positive and alkaline phosphatase interference was proved to have been the responsible for this.

## 1 INTRODUCTION

Current guidelines state that hospitalization is recommended in acute pericarditis for patients with myocardial involvement (namely myopericarditis), which is diagnosed by troponin elevation [1]. However, it should not be forgotten that elevation of cardiac troponin I, as a laboratory test, may be due to a false positive result due to a variety of causes: heterophile antibodies, autoantibodies, rheumatoid factor, fibrin clots, immunocomplex formation, interference of alkaline phosphatase or endogenous components in blood (bilirubin, hemolysis, lipids) or analyzer malfunction [2, 3].

In our institution, for practical reasons, two different troponin I assays are employed. In the emergency laboratory - which executes tests for patients in the emergency department - the Beckman Coulter Access AccuTnI+3^®^ (Marseilles, France) is used while in the routine laboratory - that executes tests for inpatients - the Abbott Architect STAT high sensitive TnI^®^ (Wiesbaden, Germany) is performed.

## 2 CASE REPORT

We report a case of a 18-year-old female patient with previous history of recurrent myopericarditis in 2015 and 2016. In both episodes, troponin I was elevated (maximum value 3.1 ng/mL) and slowly declined. In the last episode she took ibuprofen 600 mg every 8h for 2 weeks and colchicine 0.5 mg twice a day for 6 months. Physical activity was restricted for 6 months. Transthoracic echocardiography was normal but cardiac Magnetic Resonance Imaging (MRI) performed latter demonstrated residual fibrosis in the lateral wall of left ventricle.

The patient remained asymptomatic until June 2017 when she complained of palpitations and mild chest pain. Physical examination, Electrocardiogram (ECG) and transthoracic echocardiography were normal but troponin I was positive (1.04 ng/mL; normal range < 0.04 ng/mL) so myopericarditis was assumed and the patient was admitted

to the hospital and ibuprofen and colchicines were initiated. The next day and until discharge, troponin I remained negative and the patient was free from symptoms. Serology for virus, interferon gamma release assay and clinical or laboratory tests for autoimmune diseases were all negative. The possibility of a false positive test on admission was thought due to the immediate fall in troponin level. However, owing to the relief of symptoms with medication, myopericarditis could not be excluded and the patient was discharged on ibuprofen and colchicine at the same dose as before.

**Fig. (1):**
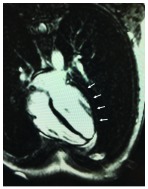
Cardiac magnetic resonance demonstrating residual fibrosis in the lateral wall of left ventricle.

Three weeks later she returned to the hospital complaining of similar symptoms. Once again, initial measurement of troponin I was positive (1.31 ng/mL) and incessant myopericarditis was assumed. Physical exam remained unremarkable as well as the ECG and transthoracic echocardiography. During hospitalization, measurements of troponin I remained always negative. She had a cardiac MRI once again but no signals of active myocarditis were detected and residual fibrosis remained as previously described (Fig. **1**). Once more, a false positive result was a possibility since the first measurements were performed by the emergency laboratory (Beckman Coulter Access AccuTnI+3^®^) and the next ones by the routine laboratory (Abbott Architect STAT high sensitive TnI^®^). To rule out a false positive result in the first measurement, performed by emergency laboratory, two samples of blood were collected at the same time and sent to both laboratories. The result was positive in the emergency laboratory (1.03 ng/mL) and negative in the routine laboratory (0.00 ng/mL). Although alkaline phosphatase activity was within the normal range (58 U/L; normal range 40-150 U/L), alkaline phosphatase interference was proved in the Beckman Coulter central laboratory. To demonstrate that, beta human chorionic gonadotropin (βhCG) was measured using the Beckman Coulter Dxl 800 Total β-hCG^®^ (Marseilles, France) assay [4] (which is also an alkaline phosphatase dependent immunoassay) and the βhCG was 1.1 IU/L (age-specific reference interval 0-0.6 IU/L [5]) while using other assay [Siemen´s Vista hCG (Erlanger, Germany)] was 0 IU/L.

Retrospectively, we confirmed that in the first two admissions, troponin I was elevated in both laboratories (and not substantially higher in one assay relatively to the other) so myopericarditis indeed existed. In order to avoid future hospital admissions, information regarding alkaline phosphatase interference with troponin I and other assays was provided to the patient and added to the patient´s files.

## 3 DISCUSSION

Currently, cardiac troponin is the most widely used cardiac biomarker in emergency departments. In the correct clinical context, troponin elevation may confirm the diagnosis of acute coronary syndromes, stratify the risk of further adverse cardiac events or result from a variety of non-coronary causes of cardiac myocyte damage or necrosis, such as myocarditis. Several immunoassays are commercially available for determination of cardiac troponin I concentrations, while only one assay for troponin T is obtainable.

The Access AccuTnI assay is a two-site immunoenzymatic “Sandwich” assay. A sample is added to a reaction vessel along with monoclonal anti-troponin I antibody conjugated to alkaline phosphatase and paramagnetic particles coated with monoclonal anti-troponin I antibody. Human troponin I binds to the anti-troponin I antibody on the solid phase, while the anti-troponin I antibody- alkaline phosphatase conjugate reacts with different antigenic sites on the troponin I molecules. After incubation, the chemiluminescent substrate Lumi-Phos* 530 is added and light generated by the reaction is measured with a luminometer. The light production is directly proportional to the concentration of troponin I in the sample [6-8]. The Abbott Architect STAT high sensitive assay is a two-step, double-monoclonal chemiluminescent microparticle immunoassay that uses a mouse-human monoclonal anti-cTnI acridinium-labeled detection antibody [9]. Serum components may interfere with these processes, leading to inaccurate quantification. Reports suggest that the AccuTnI+3 assay has a higher frequency of false elevations than other troponin I tests [7-10]. As the Beckman Coulter Access AccuTnI+3^®^ uses alkaline phosphatase for signal amplification while the Abbott Architect STAT high sensitive TnI^®^ uses acridinium, the first suspicion was interference of alkaline phosphatase. In fact, elevated alkaline phosphatase can interfere with contemporary alkaline phosphatase-dependent immunoassays as recently demonstrated [4] but this is not the case in our report since alkaline phosphatase was normal. In the same study [4], it is speculated that endogenous alkaline phosphatase associated with microparticles may be responsible for some non-reproducible false elevations. The authors consider this is the most probable hypothesis in our patient.

Unfortunately, since there are the many manufacturers of troponin I assays, the prevalence of clinically significant inaccurate quantification of troponin I is unknown [11]. Given its extensive use, false positive results could result in harm to patients and/or avoidable costs (unnecessary non-invasive exams, invasive procedures, hospital admissions). Therefore, clinical evaluation is always necessary to avoid incorrect diagnosis. In our case, the patient had been previously diagnosed with acute myopericarditis and cardiac MRI confirmed it. Since recurrent pericarditis is frequent, even in patients who took colchicine, this diagnosis seemed reasonable in the presence of positive troponin I, despite atypical chest pain and normal ECG and transthoracic echocardiography. However, suspicion of a false positive result was obvious during the second admission.

The authors demonstrate that a false positive troponin I test may lead to inaccurate diagnosis with important clinical implications, if clinical judgment is not used. In this case, two hospital admissions were not necessary; in other situations, as acute coronary syndrome suspicion, a false positive result lead in an unnecessary angiography [12, 13]. As far as the authors know, this is a unique case since alkaline phosphatase interference occurred in a patient with alkaline phosphatase in the normal range. The authors suggest that for laboratories using the AccuTnI+3 assay, even in patients with normal values of alkaline phosphatase, interference can occur and should be excluded when clinically appropriate. Fortunately, more recent troponin I assays, although not free from false positive results, are less prone to give rise to this error. Indeed, limitations of any chosen test for troponin I measurement must be known to all physicians dealing with it.
